# Quality Analysis of Online Resources for Patients Undergoing Coronary Artery Bypass Grafting

**DOI:** 10.1016/j.atssr.2023.12.021

**Published:** 2024-02-01

**Authors:** Natalia Roa-Vidal, John A. Treffalls, Zachary Brennan, Omar M. Sharaf, Brittany Rhoades, Lauren K. Barron

**Affiliations:** 1 School of Medicine, University of Puerto Rico Medical Science Campus, San Juan, Puerto Rico; 2 Long School of Medicine, University of Texas Health San Antonio, San Antonio, Texas; 3 Michigan State University College of Osteopathic Medicine, Michigan State University, East Lansing, Michigan; 4 Division of Cardiovascular Surgery, Department of Surgery, University of Florida Health, Gainesville, Florida; 5 Division of Cardiothoracic Surgery, Michael E. DeBakey Department of Surgery, Baylor College of Medicine, Houston, Texas; 6 The Texas Heart Institute, Houston, Texas

## Abstract

**Background:**

Online resources are becoming the primary educational resource for patients. Quality and reliability of websites about coronary artery bypass graft (CABG) procedures are unknown.

**Methods:**

We queried 4 search engines (Google, Bing, Yahoo!, and Dogpile) for the terms *coronary artery bypass*, *coronary artery bypass graft*, *coronary artery bypass graft surgery*, and *CABG*. The top 30 websites from each were aggregated. After exclusions, 85 websites were graded with the DISCERN instrument, patient-focused criteria, and readability calculators by a 2-reviewer system.

**Results:**

Accessibility was low; 34.1% of websites disclosed authorship, and 23.5% were available in Spanish. Median total score was 55 of 95 (interquartile range [IQR], 44-68); this score varied by website type (*P* = .048). Professional medical society (median, 76; IQR, 76-76) and governmental agency (median, 69; IQR, 56.6-75.5) scored higher, whereas industry (median, 51.8; IQR, 47.1-56.4) and hospital/health care (median, 49; IQR, 40-61) scored lower. Readability was low, with median Flesch-Kincaid grade level score of 11.1 (IQR, 9.5-12.6) and 75.3% of websites written above eighth-grade reading level.

**Conclusions:**

Accessibility of online patient educational resources for CABG procedures is limited by language and reading level despite being widely available. Quality and reliability of the information offered varied between website types. Improving readability to ensure patients’ understanding and comprehensive decision-making should be prioritized.

**In Short**

▪ Quality could be augmented with broader explanations of complications and alternative options.
▪ Increasing authorship and source disclosures could improve reliability.
▪ Websites could be more accessible by adding translation services.

Coronary artery disease (CAD) is the leading cause of death in adults and Hispanics in the United States.^1^ Higher incidence of CAD among Hispanics may be explained by high incidence of obesity, sedentary life style, smoking, and socioeconomic disparities.^1^ Management of CAD may involve medical therapy, percutaneous coronary intervention, or coronary artery bypass graft (CABG) surgical operation. For patients considering CABG procedures, understanding of potential complications and postoperative expectations is essential.

Increased access to online resources has prompted patients to research medical symptoms, conditions, and procedures. Moreover, online information has the ability to influence patients’ medical decision-making.^2^ This highlights the importance of physicians’ providing adequate education about conditions and procedures in addition to guiding patients to accurate and comprehensible online resources.

Many online patient educational resources are written above an eighth-grade reading level, the average reading level in the United States.^3^ Studies examining online information for peripheral artery disease and heart failure consistently concluded that online information demands patients to have high health literacy.^4^^,^^5^ A systematic review conducted in 2020 assessing online information on CABG and percutaneous coronary intervention found that websites required college-level reading comprehension.^6^ Approximately 90 million US adults are considered to have limited health literacy, with higher rates among the elderly, economically disadvantaged, and minorities, predisposing these populations to poorer health outcomes.^7^ As use of online resources increases as a source of health education,^2^ it is imperative to evaluate the accessibility of current resources. We aimed to systematically analyze available internet resources for English- and Spanish-speaking patients considering CABG procedures.

## Material and Methods

Internet searches for the terms *coronary artery bypass*, *coronary artery bypass graft*, *coronary artery bypass graft surgery*, and *CABG* were completed in December 2022 using incognito Chrome browser to decrease the impact of browser data. Search engines queried were Google, Bing, Yahoo!, native search engines, and Dogpile (a metasearch engine). The first 30 websites from each term on each search engine were compiled in order of appearance to select the most relevant. Websites (n = 158) were reviewed to exclude duplicates, commercial websites, physician-oriented resources, and broken links for a final list of 85 websites.

A 2-reviewer system scored websites. Reviewers (N.R.V., J.A.T., Z.B., O.M.S.) were trained on the scoring systems and criteria approved by a trained coronary surgeon. For the assessment of websites’ reliability and quality, DISCERN—a validated quality criterion for consumer health information—was employed. It is composed of questions that assess 2 components: reliability (ie, aim, references, recentness, and additional sources) and content (ie, description of treatment, risks, benefits, and alternatives). To address website accessibility and interactivity, the original assessment tool created by Ingledew^8^ was adopted. To evaluate readability, standardized scoring scales (Flesch-Kincaid, Coleman-Liau, Automated Readability Index) were used through open-access websites. A detailed description of the scoring criteria can be found in the Supplemental Table. For all categories, scores from reviewers were averaged, with discrepancies resolved by consensus. Data were analyzed by Jamovi 2.3.21. Kruskal-Wallis nonparametric analysis of variance was performed to assess differences between website types. Nonparametric statistical tests were used as most of the data analyzed were nonnormal, defined as a Shapiro-Wilk normality test *P* > .05. Dwass-Steel-Critchlow-Fligner tests were performed post hoc to assess pairwise differences. Video-based websites were assessed for content by DISCERN but were excluded from readability analysis. Statistical significance was defined as *P* ≤ .05.

## Results

### Websites

After exclusions, 85 websites remained. Website types included 43 hospital/health care organization (50.6%), 30 open access (35.3%), 9 governmental agency (10.6%), 2 industry sponsored (2.4%), and 1 professional medical society (1.2%). Table 1 displays the top 3 websites in content, in readability, and overall.

**Table 1:** Top Online Resources for Patients Undergoing CABG Procedures

Website URL	Score	Website Type
DISCERN total score		
https://www.encyclopedia.com/medicine/divisions-diagnostics-and-procedures/medicine/coronary-artery-bypass-graft-surgery	77.50	Open access
https://en.wikipedia.org/wiki/Coronary_artery_bypass_surgery	76.50	Open access
https://www.verywellhealth.com/heart-bypass-surgery-overview-5087616	76.00	Open access
DISCERN quality		
https://www.encyclopedia.com/medicine/divisions-diagnostics-and-procedures/medicine/coronary-artery-bypass-graft-surgery	33.50	Open access
https://www.verywellhealth.com/heart-bypass-surgery-overview-5087616	33.50	Open access
https://www.bhf.org.uk/informationsupport/treatments/coronary-bypass-surgery	33.50	Open access
DISCERN reliability		
https://www.encyclopedia.com/medicine/divisions-diagnostics-and-procedures/medicine/coronary-artery-bypass-graft-surgery	39.00	Open access
https://en.wikipedia.org/wiki/Coronary_artery_bypass_surgery	38.50	Open access
https://www.verywellhealth.com/coronary-artery-bypass-graft-cabg-5088620	38.50	Open access
Readability (Flesch-Kincaid reading level)		
https://www.drugs.com/cg/cabg-coronary-artery-bypass-graft.html	5.6	Open access
https://healthy.kaiserpermanente.org/health-wellness/health-encyclopedia/he.coronary-artery-bypass-graft-what-to-expect-at-home.ud1743	5.9	Health care
https://myhealth.alberta.ca/Health/aftercareinformation/pages/conditions.aspx?hwid=ud1743	6.0	Government

CABG, coronary artery bypass graft; DISCERN, a validated quality criterion for consumer health information.

### Quality

**Figure 1:**
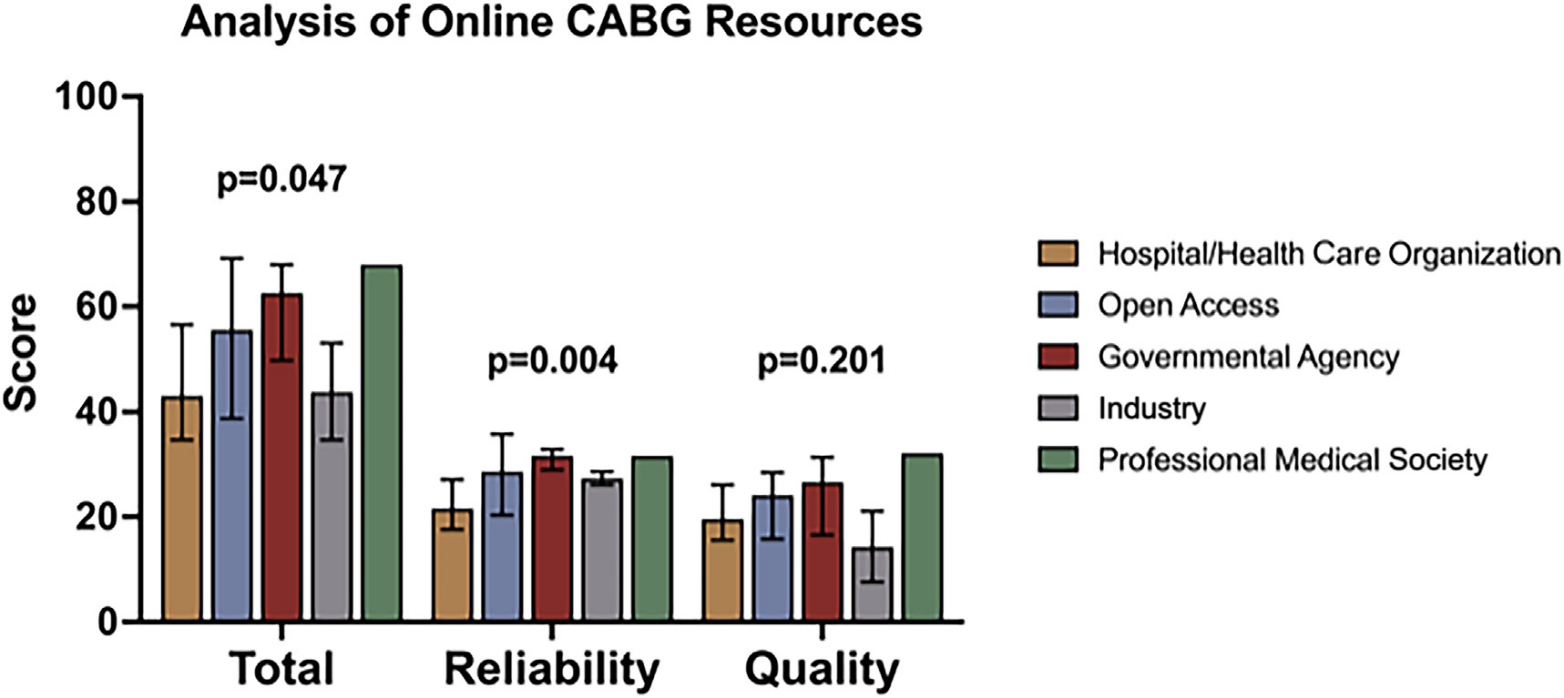
Analysis of online patient resources for coronary artery bypass graft (CABG) procedures by the DISCERN criteria (DISCERN, a validated quality criterion for consumer health information.).

The quality of online resources for CABG patients was variable, with a total score range of 69 (18.5-87.5). Median total score for all 85 websites was 58.4% (55.5/95; interquartile range [IQR], 44.0-68.5). The total DISCERN score, composed of 3 variables (reliability, quality, and subjective overall quality), was 62.5% (50/80; IQR, 39.0-61.5); reliability was 60% (24/40; IQR, 19.5-31.5), quality was 58.6% (20.5/35; IQR, 16.0-27.5), and subjective overall quality was 60% (3/5; IQR, 2-4; Figure 1; Table 2). Accessibility was 33.3% (1/3; IQR, 1-2), and interactivity was 25% (3/12; IQR, 3-6). Only 23.5% of websites were available in Spanish. Authorship was disclosed by 34.1% of websites, and 18.8% included references. Total score varied by website type (*P* = .047; Figure 1), with the professional medical society website (80%; median, 76/95; IQR, 76-76) and governmental websites (72.1%; median, 68.5/95; IQR, 55.5-74.0) scoring highest and hospital/health care organization websites scoring lowest (51.6%; median, 49/95; IQR, 40-61). However, on pairwise comparison, there were no differences by website type (all *P* < .05). This difference was primarily driven by variation in the DISCERN reliability score (*P* = .004), which displayed a similar trend. On pairwise comparison, government and hospital/health care organization websites were different (*P* = .005). There was a trending difference in open access and hospital/health care organization websites (*P* = .063). All other differences were nonsignificant. The DISCERN quality score did not vary by website type (*P* = 0.2).

**Table 2:** Online Resource Quality Analysis for CABG Procedures by Website Type

Variable	Hospital/Health Care (n = 43)	Open Access (n = 30)	Governmental Agencies (n = 9)	Industry (n = 2)	Professional Medical Society (n = 1)	*P* Value
Total score (of 95)	49 (40.0-61.0)	61.5 (43.4-76.8)	69 (56.6-75.5)	51.75 (47.1-56.4)	76.0 (76.0-76.0)	.05
DISCERN total (of 80)	43 (35.3-55.8)	55.5 (39.9-68.5)	62.5 (50.4-67.6)	43.75 (39.1-48.4)	68.0 (68.0-68.0)	.02
DISCERN reliability (of 40)	21.5 (17.8-26.5)	28.5 (20.6-35.3)	31.5 (29.9-32.3)	27.25 (26.6-27.9)	31.5 (31.5-31.5)	.004
DISCERN quality (of 35)	19.5 (15.8-25.3)	24 (16.1-27.9)	26.5 (18.1-30.9)	14.25 (10.9-17.6)	32.0 (32.0-32.0)	.2
Accessibility (of 3)	1 (1.0-2.0)	2 (1.0-2.0)	2 (1.0-2.0)	2 (1.5-2.5)	3.0 (3.0-3.0)	.19
Interactivity (of 12)	4 (3.0-5.0)	3 (2.0-6.8)	5 (3.3-7.3)	6 (5.5-6.5)	5 (5.0-5.0)	.37
Flesch-Kincaid grade level	11.7 (10.1-13.0)	10.7 (9.1-12.6)	10.15 (8.4-11.3)	12.65 (10.4-14.9)	11 (11.0-11.0)	.30
Coleman-Liau	12 (10.5-12.0)	12 (10.0-12.2)	10 (10.0-10.0)	12.5 (10.8-14.2)	10.5 (10.5-10.5)	.08
Automated Readability Index	11 (9.7-12.1)	10.1 (9.0-11.8)	9.8 (8.8-10.5)	11.55 (9.7-13.4)	11.2 (11.2-11.2)	.14

CABG, coronary artery bypass graft; DISCERN, a validated quality criterion for consumer health information.

Most websites mentioned what a CABG procedure comprises (84.7%); however, only 44.7% explained different procedural considerations, such as graft choices or surgical strategy (ie, on-pump vs off-pump). Few websites (12.9%) explained benefits of CABG procedures in 3 or more sentences, and 30.6% abounded in expectations after surgical operation, quality of life, or medical treatment after surgical operation. Less than one-third (31.8%) discussed surgical risks in 3 or more sentences detailing procedural complications, short-term risks, and long-term risks. Only 17.6% of websites mentioned CABG alternatives for patients who might be candidates for these options.

### Readability

**Figure 2:**
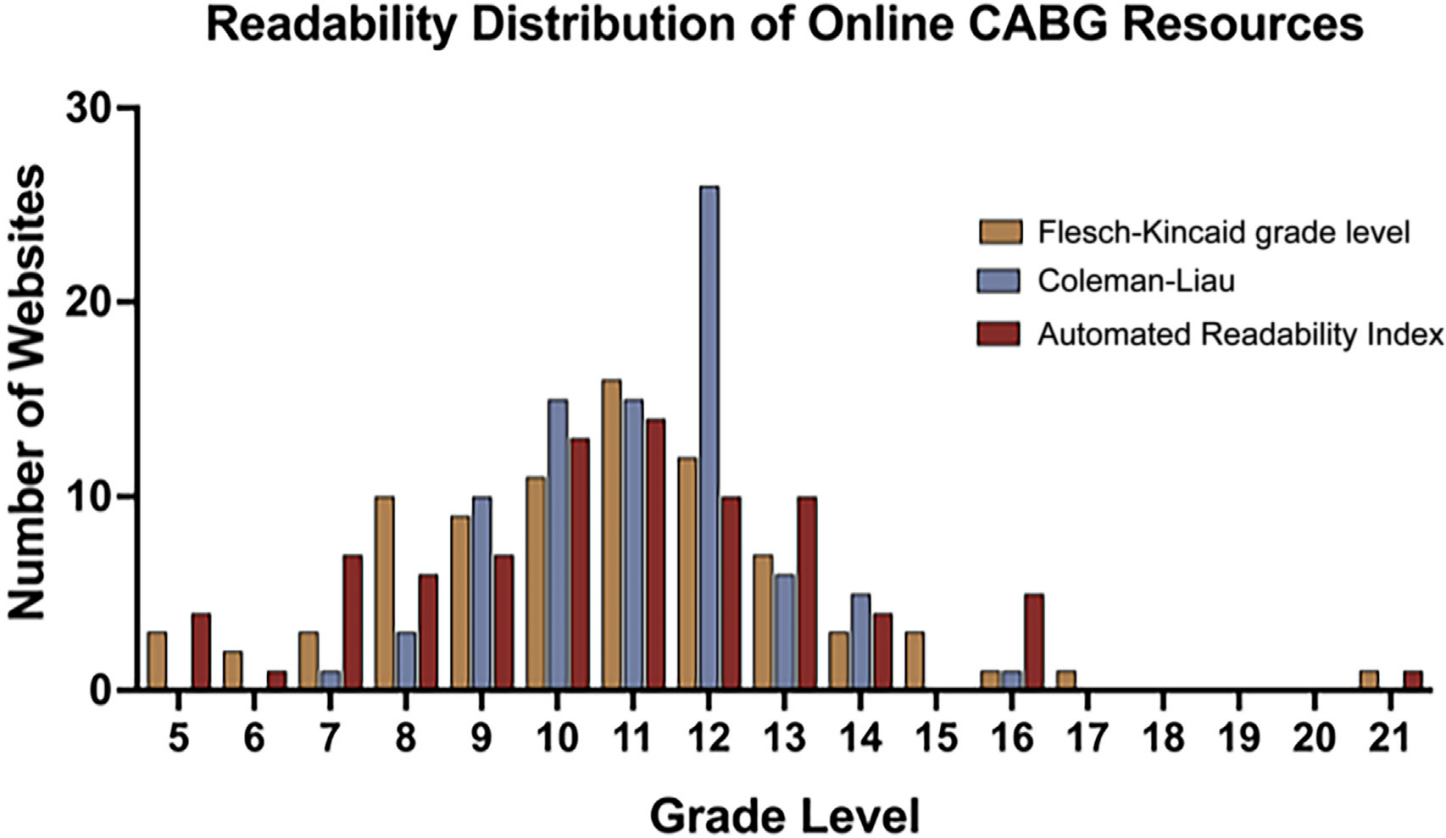
Distribution of readability scores of online patient resources for coronary artery bypass graft (CABG) procedures by Flesch-Kincaid grade level, Coleman-Liau, and Automated Readability Index formulas.

Of 85 websites evaluated, 3 video-based websites were excluded from the readability analysis. Readability of the 82 included websites was low, with a median Flesch-Kincaid grade level of 11th grade (IQR, 10th-13th-grade; Figure 2), similar across website types (*P* = .3; Figure 2). Automated Readability Index (median, 11th grade; IQR, 10th-13th grade; *P* = .14) and the Coleman-Liau index (median, 11th grade; IQR, 10th-12th grade; *P* = .09) resulted in similar readability. With use of the Flesch-Kincaid grade level, only 22% (18/82) of websites were written at or below an eighth-grade reading level, with 19.5% (16/85) of websites written at a college or higher reading level.

## Comment

Our analysis of available internet resources for English- and Spanish-speaking patients considering a CABG procedure revealed that overall quality was low, differing by website type, with hospital/health care organization websites scoring the lowest. Less than one-third of websites discussed procedural complications, short-term risks, or long-term risks in 3 or more sentences, demonstrating a need for improvement. Furthermore, few online resources thoroughly detailed benefits of CABG, presenting a clear opportunity for websites to offer an introduction to the risk/benefit ratio for different populations of patients as well as how patients can ask their medical team to help understand individualized risk calculations. In addition, less than one-fifth of websites mentioned alternative treatment options. Given the importance of discussing all available options to encourage shared decision-making, patient-centered websites may improve by discussing treatment alternatives and the circumstances in which a patient would be a candidate.

Low accessibility and reliability scores were influenced by lack of authorship disclosure and citation of sources as most websites did not specify authorship, reflecting a need of increased transparency to promote reliability and credibility of online resources for CABG patients. Accessibility could also be increased by expanding language options; less than one-quarter of websites were available in Spanish. Hispanic populations in the United States are highly affected by CAD,^1^ and in this primarily Spanish-speaking population, decision-making is undoubtedly affected by limited English literacy. Language barriers in addition to unfamiliarity with medical terminology and complex procedures only increase the disparities experienced by this population of patients.^9^ Providing alternatives in native languages may increase comprehension and security in decision-making when a CABG procedure is being considered. Websites should consider adding translation services (ie, Google Translate), videos, and diagrams to offer alternative communication strategies to relay health information.

Decreased health literacy, defined as ability to understand health information required to make informed decisions, is a factor in the perpetuation of health inequality.^1^ Patients with difficulty in understanding health information have 1.4-fold higher risk of death after 6 years,^10^ emphasizing the importance of patient-accessible online resources as the average person in the United States reads at a seventh- or eighth-grade level.^3^ Our study showed that most information about CABG is written at or above the 11th-grade level; less than one-quarter is written at an eighth-grade level. Addressing this disparity may improve patients’ understanding of CAD and treatment options, which may help ensure that patients have adequate capacity to make informed decisions.

This study has important limitations. We used standardized DISCERN criteria and a 2-reviewer system to assess websites objectively, but it is possible that reviewer subjectivity affected the results. Our survey of websites is cross-sectional, and online content constantly changes. Future search results could be influenced by location and user search history.

Online patient resources for CABG had low reliability, quality, and readability, highlighting the need and offering focused directions for improvements. Increasing sources and authorship disclosure would offer transparency and reliability, enhancing patients’ trust in the resources they are accessing. In terms of content, websites should offer more in-depth explanations of the procedure and associated risks. Finally, increasing the number of Spanish available websites or incorporating artificial intelligence–assisted translation services where patients choose the language of the content (button at bottom left corner at https://www.texasheartmedical.org/) could help close the gap on the language barrier that Hispanic patients face in accessing health education.
