# Supplementary material for: Quality Analysis of Online Resources for Patients Undergoing Coronary Artery Bypass Grafting
Source: Ann Thorac Surg Short Rep. 2024 Feb 1;2(3):331–5. doi: 10.1016/j.atssr.2023.12.021 (PMC11708284; doi:10.1016/j.atssr.2023.12.021)
Supplement: Supplemental Table [file mmc1.docx]

**Supplementary Table 1.** Website Quality Assessment Questions and Scoring

| **DISCERN: Reliability** | | |
| --- | --- | --- |
|  | Are the aims clear (i.e orients patients about CABG)? | |
|  |  | Yes 🡪 5  Partial 🡪 2-4  No 🡪 1 |
|  | Does it achieve its aims (i.e. explains the procedure, mentions risks and benefits)? | |
|  |  | Yes 🡪 5  Partial 🡪 2-4  No 🡪 1 |
|  | Is it relevant (i.e, addresses the questions that readers might ask and are treatment recommendations realistic or appropriate)? | |
|  |  | Yes 🡪 5  Partial 🡪 2-4  No 🡪 1 |
|  | Is it clear what sources of information were used to compile the publication, other than the author or producer (i.e main claims or statements made about treatment choices are accompanied by a reference to the sources used as evidence and there are means available of for checking sources used)? | |
|  |  | Yes 🡪 5  Partial 🡪 2-4  No 🡪 1 |
|  | Is it clear when the information used or reported in the publication was produced (i.e. dates of main sources of information used, of any revisions and publication)? | |
|  |  | Yes 🡪 5  Partial 🡪 2-4  No 🡪 1 |
|  | Is it balanced and unbiased (i.e. presents a clear indication of whether the publication is written from a personal or objective point of view using a wide range of sources of information and that an external assessment completed)? | |
|  |  | Yes 🡪 5  Partial 🡪 2-4  No 🡪 1 |
|  | Does it provide details of additional sources of support and information? (i.e. suggests further reading)? | |
|  |  | Yes 🡪 5  Partial 🡪 2-4  No 🡪 1 |
|  | Does it refer to areas of uncertainty (i.e. mentions gaps in knowledge or differences in expert opinion regarding treatment choices)? | |
|  |  | Yes 🡪 5  Partial 🡪 2-4  No 🡪 1 |
| **DISCERN: Quality of information of treatment** | | |
|  | Does it describe how CABG is done (i.e explains in detail, mentions different approaches and techniques)? | |
|  |  | Yes (≥3 sentences) 🡪 5  Partial (1-2 sentences)🡪 2-4  No 🡪 1 |
|  | Does it describe the benefits of CABG? | |
|  |  | Yes (≥3 sentences) 🡪 5  Partial (1-2 sentences)🡪 2-4  No 🡪 1 |
|  | Does it describe the risks of CABG? | |
|  |  | Yes (≥3 sentences) 🡪 5  Partial (1-2 sentences)🡪 2-4  No 🡪 1 |
|  | Does it describe what would happen if CABG was not done in a patient that needs it (i.e. describes risks and benefits of postponing treatment or permanently forgoing treatment)? | |
|  |  | Yes (≥3 sentences) 🡪 5  Partial (1-2 sentences)🡪 2-4  No 🡪 1 |
|  | Does it describe how CABG affect overall quality of life (i.e. what to expect of life after CABG, recovery, suggested lifestyle changes, medical treatment post-surgery)? | |
|  |  | Yes (≥3 sentences) 🡪 5  Partial (1-2 sentences)🡪 2-4  No 🡪 1 |
|  | Is it clear that there may be more than one possible treatment choice (i.e. Medical Therapy, PCI, MIDCAB and who might be a candidate for each treatment)? | |
|  |  | Yes (≥3 sentences) 🡪 5  Partial (1-2 sentences)🡪 2-4  No 🡪 1 |
|  | Does it provide support for shared decision-making (i.e suggests things to discuss with family, friends, doctors, other health professionals)? | |
|  |  | Yes 🡪 5  Partial 🡪 2-4  No 🡪 1 |
| **DISCERN: Overall Quality** | | |
|  | Based on the answers to all of the above questions, rate the overall quality of the publication as a source of information about treatment choices. | |
|  |  | High: rated high (≥4) in majority of questions 🡪 5  Moderate: rated high and low on similar number of questions or majority in mid-ranges🡪 3  Low: rated low (≤2) in majority of questions 🡪 1 |
| **Accessibility** | | |
|  | Does link lead directly to info on CABG? | |
|  |  | Yes 🡪 1  No 🡪 0 |
|  | Is the author/editor clearly identifiable? | |
|  |  | Yes 🡪 1  No 🡪 0 |
|  | Available in Spanish? | |
|  |  | Yes 🡪 1  No 🡪 0 |
| **Interactivity** | | |
|  | How many external links are present (must be to websites of a different affiliation)? | |
|  |  | ≥2 🡪 2  1 🡪 1  None 🡪 0 |
|  | Are external links functional? | |
|  |  | ≥50% 🡪 2  <50% 🡪 1  None 🡪 0 |
|  | Is within-site search available? | |
|  |  | Yes 🡪 1  No 🡪 0 |
|  | Is there audio or video support? | |
|  |  | Yes 🡪 1  No 🡪 0 |
|  | Are there patient discussion boards or forums? | |
|  |  | Yes 🡪 1  No 🡪 0 |
|  | Is there a phone number or ability to message/email? | |
|  |  | Yes 🡪 1  No 🡪 0 |
|  | Does it contain diagrams, pictures, tables (must be within the body of text and related to CABG)? | |
|  |  | Yes 🡪 1  No 🡪 0 |
|  | Does it contain hyperlinks (Must be within the body of text)? | |
|  |  | Yes 🡪 1  No 🡪 0 |
|  | Is there absence of advertising? | |
|  |  | Yes 🡪 1  No 🡪 0 |
|  | Is the advertisement policy disclosed? If no advertisement answer 0 | |
|  |  | Yes 🡪 1  No 🡪 0 |
| **Readability Tests Link** | | |
|  | Flesch-Kincaid, Coleman-Liau, Linsear Write, Automated Readability Index | |
|  |  | <https://www.readabilityformulas.com/free-readability-formula-tests.php> |
